# MicroRNAs Expression Profiles in Cardiovascular Diseases

**DOI:** 10.1155/2014/985408

**Published:** 2014-06-12

**Authors:** Elsa Bronze-da-Rocha

**Affiliations:** ^1^Departamento de Ciências Biológicas, Laboratório de Bioquímica, Faculdade de Farmácia da Universidade do Porto, Rua Jorge Viterbo Ferreira 228, 4050-313 Porto, Portugal; ^2^Instituto de Biologia Molecular e Celular da Universidade do Porto, Rua do Campo Alegre 823, 4150-180 Porto, Portugal

## Abstract

The current search for new markers of cardiovascular diseases (CVDs) is explained by the high morbidity and mortality still observed in developed and developing countries due to cardiovascular events. Recently, microRNAs (miRNAs or miRs) have emerged as potential new biomarkers and are small sequences of RNAs that regulate gene expression at posttranscriptional level by inhibiting translation or inducing degradation of the target mRNAs. Circulating miRNAs are involved in the regulation of signaling pathways associated to aging and can be used as novel diagnostic markers for acute and chronic diseases such as cardiovascular pathologies. This review summarizes the biogenesis, maturation, and stability of miRNAs and their use as potential biomarkers for coronary artery disease (CAD), myocardial infarction (MI), and heart failure (HF).

## 1. Introduction 

Aging is a gradual and multidimensional process of physical, psychological, and social changes. Different degrees of molecular and cellular modifications may lead to a variety of health challenges in an individual and may play a key role in the development of aging and age-related diseases such as cardiovascular and neurodegenerative diseases, immune disorders, and cancer [1–3]. Cardiovascular diseases (CVD) are the major cause of worldwide death, particularly in the elderly population presenting an increasing rate of mortality and morbidity, and they are a consequence of genetic and epigenetic interactions [1–3]. The genetic components include the genomic instability, cellular senescence, telomere lengthening, signaling network, dietary restriction, molecular damage, in particular oxidative injury, overactivity during adulthood of processes that can lead to hypertrophy-associated pathologies (hyperfunction), loss of proteostasis, mitochondrial dysfunction, stem cell exhaustion, and alterations in the intercellular communication [4–8]. The most important epigenetic modifications of mammalian cells are associated to DNA methylation, posttranslational histone modifications, and to a class of short noncoding RNAs, the microRNAs (miRNAs or miRs) [6, 9]. microRNAs are key components of many cellular processes. Different studies have demonstrated that miRNA expression is tissue-specific, tightly regulated during embryogenesis, and overexpressed/underexpressed in many diseases, including cardiovascular pathologies [10, 11]. Presently, most of the studies are investigating the utility of individual miRNAs or patterns of multiple miRNAs as biomarkers for diseases and the use of antagomirs and miRNAs mimics to restore the miRs expression levels.

In this review, the biogenesis and processing of miRNAs, as well as their release, stability, and modulation, will be addressed. Regarding the miRNAs expression profiles, the current potential biomarkers for some human heart diseases will be summarized and discussed.

## 2. The Biogenesis, Maturation, and Nomenclature of miRNAs

microRNAs are a conserved class of small noncoding RNAs (ncRNAs) endogenously produced that regulate gene expression at the posttranscriptional level in both physiological and disease conditions. miRNAs have a function in cell proliferation, differentiation, metabolism, apoptosis, development, and aging and in the pathophysiology of many diseases, namely, in oncogenesis, cardiovascular, and neurological disorders [12–15]. They were originally found in the nematode* Caenorhabditis elegans* (*C. elegans*) in 1993 [16] and later identified in many plants and mammals [17]. Modulation of miRNA expression* in vitro* as well as* in vivo* has revealed an important role for miRNAs in the regulation of heart function, particularly cardiac growth, hypertrophy, and failure [18].

The miRNA coding regions are present in the genome as clusters transcribed as polycistronic primary transcripts, independent units, like intergenic regions, and within introns of protein coding or noncoding sequences [19]. The classical miRNA production is named the canonical or miRNA pathway. In general, miRNAs are transcribed in the nucleus by the RNA polymerase II [20, 21] as a pri-miRNA, a primary transcript of several hundred nucleotides in length, bearing a hairpin-shaped structure that temporarily receive a 5′-cap and a 3′-poly(A) tail. A small group of miRNA genes are also transcribed by RNA polymerase III [21, 22]. Pri-miRNAs are cleaved into approximately 70-nucleotide precursor-miRNAs, the pre-miRNAs, by the microprocessor complex that contains a RNAse III enzyme called Drosha and its cofactor DiGeorge syndrome critical gene 8 (DGCR8) [23]. The pre-miRNAs are then exported to the cytoplasm by exportin-5 (Exp 5), where they are further cleaved into mature 20–25 nucleotide miRNA duplexes by another RNAse III endonuclease, called Dicer, and by its double stranded RNA binding cofactor TAR RNA binding protein (TRBP, also called loquacious, Loqs), among other proteins [24]. In the cytoplasm, the mature miRNA duplexes are separated into two RNA strands, the guide RNA strand (miRNA) and the passenger RNA strand (miRNA*) which is usually degraded. The RNA-induced silencing complex (RISC), containing the argonaute (Ago) proteins, is activated by the presence of the miRNA and directs the miRNA-induced silencing complex (miRISC) to the target mRNA [24–26]. The miRNA target site is often present in the 3′-untranslated region (3′-UTR) of the mRNA [23] that contains complementary sequences, designated miRNA recognition elements (MREs). According to the perfect or imperfect complementarity of the miRNA-mRNA sequences, miRNAs can repress gene expression by either inducing mRNA degradation or by blocking translation [17, 27–29]. It has been reported that miRNAs can also bind to the 5′-untranslated region (5′-UTR) [30] or to the open reading frame (ORF) [31]. However, endogenous ORF targeting seems to be less frequent and effective than 3′-UTR targeting but still more frequent than 5′-UTR targeting [28]. Prevention of protein translation could be achieved by: deadenylation of the poly(A) tail; competition of the Ago-RISC complex with translation initiation factors and cap structure; blocking translation elongation; promoting premature dissociation of ribosomes; and degradation of the nascent polypeptide chain [32, 33].

**Figure 1:**
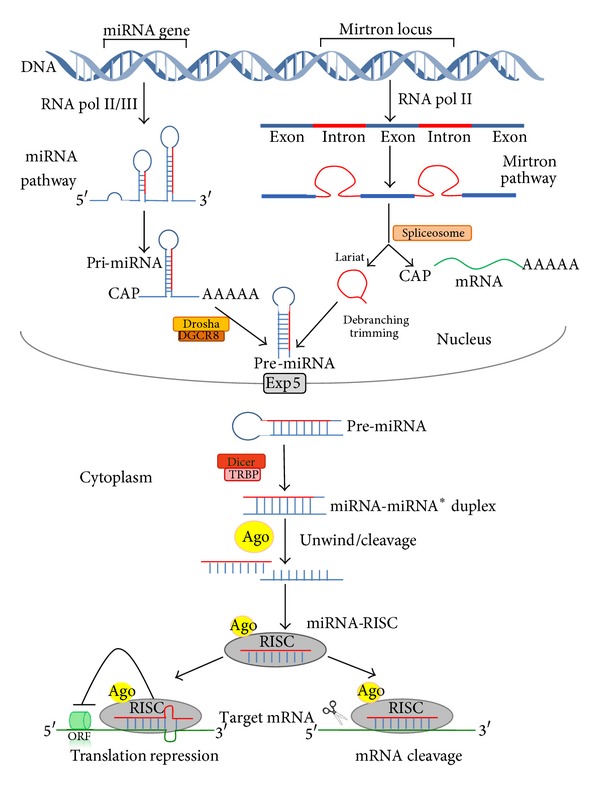
The miRNA or canonical pathway produces pri-miRNA transcripts from miRNA genes by RNA polymerase II or III (RNA pol II/III) followed by Drosha/DGCR8 processing of the pri-miRNA transcripts into pre-miRNAs. Intronic pre-miRNA hairpins of the mirtron or noncanonical pathway, transcribed by RNA polymerase II (RNA pol II), are formed by splicing (spliceosome), debranching, and trimming of short introns (lariat) without Drosha processing. Pre-miRNAs generated by both pathways are exported from the nucleus via exportin-5 (Exp 5), followed by subsequent Dicer/TRBP which generates double-stranded-RNAs called miRNA/miRNA*. Argonaute proteins (Ago) unwind and separate the guide strand (miRNA) and the passenger strand (miRNA*). The RISC (RNA-induced silencing complex) incorporates the mature miRNA and interacts with the 3′-UTR of the target mRNA and regulates gene expression by translation inhibition or mRNA degradation.

An alternative nuclear miRNA biogenesis pathway, the mirtron or microprocessor-independent pathway or noncanonical pathway, includes a group of short introns, termed mirtrons which has been described in invertebrates and mammals [34]. Mirtron biogenesis requires the spliceosomal machinery and is initiated by splicing and debranching into a pre-miR hairpin, which is suitable for Dicer cleavage and is incorporated into silencing complexes [34–36]. This intron-derived miRNA is not only able to induce RNA interference (RNAi) in mammalian cells, but also in fish, chicken embryos, and adult mice, indicating the evolutionary conservation of this mechanism of gene regulation* in vivo* [37, 38]. Some small nucleolar RNAs (snoRNAs) also provide a secondary source of pre-miRNAs that is independent of the microprocessor-mediated processing. Additionally, miRNAs can also derive from endogenous short-hairpin RNAs (endo-shRNAs) and tRNA precursors [35]. A third pathway for the production of miRNAs, the Dicer-independent pathway, was recently identified in zebrafish and mammals [15].  Figure 1 shows a schematic overview of the miRNA and mirton pathways.

miRNAs are identified using a combination of criteria for both expression and biogenesis [39]. Names are referred to by the miRNA Register [40] and numerical identity is based on sequence similarity [41]. The genes that encode the miRNA are also named using the same three-letter prefix, with capitalization, hyphenation, and italics according to the conventions of each organism (e.g.,* mir-1* in* C. elegans* and* Drosophila* and* MIR156* in* Arabidopsis* and rice). Plant and viral naming schemes differ subtly [41]. The identifying numbers are assigned sequentially, with identical miRNAs having the same number, regardless of the organism [41]. The database uses abbreviated three or four letter prefixes to designate the species, such that identifiers take the form hsa-miR-101 (in* Homo sapiens*) [41]. The “mir” is followed by a dash and a number, the latter often indicating order of naming. For example, mir-318 was named and discovered earlier than mir-319. The “mir-” refers to the pre-miRNA, while a capitalized “miR-” refers to the mature form. Pre-miRNAs that lead to 100% identical mature miRNAs but that are located at different places in the genome are indicated with an additional dash-number suffix. For example, the pre-miRNAs hsa-mir-194-1 and hsa-mir-194-2 lead to an identical mature miRNA (hsa-miR-194) but are located in different regions of the genome [39, 41]. miRNAs with nearly identical sequences except for one or two nucleotides are annotated with an additional lower case letter. For example, miR-123a would be closely related to miR-123b [39, 41]. Identical or very similar miRNA sequences within a species can also be given the same number, with their genes distinguished by letter and/or numeral suffixes, according to the convention of the organism; for example, the ∼22-nt transcripts of* Drosophila mir-13a* and* mir-13b* are slightly different in sequence, whereas those of* mir-6*-*1* and* mir-6*-*2* are identical [39]. The gene names are planned to convey limited information about functional relationships between mature miRNAs. For example, hsa-miR-101 in human and mmu-miR-101 in mouse are orthologous. Paralogous sequences whose mature miRNAs differ at only one or two positions are given lettered suffixes, for example, mmu-miR-10a and mmu-miR-10b in mouse [41]. Two different mature miRNA sequences which were excised from opposite arms of the same hairpin precursor are currently named as miR-17-5p (5′ arm) and miR-17-3p (3′ arm). When relative expression levels are known, an asterisk following the name indicates a miRNA expressed at low levels relative to the miRNA in the opposite arm of a hairpin. For example, miR-123 and miR-123* would share a pre-miRNA hairpin, but more miR-123 would be found in the cell [39, 41].

## 3. Stability of Circulating miRNAs

Accumulation of a specific miRNA is dependent on the rates of transcription, processing, and decay. The expression of miRNAs transcripts is regulated by specific transcription factors and dependent on the methylation of their promoter sequences in the same way as the expression of many protein-coding genes [24, 42].

The availability of argonaute proteins affects the miRNAs abundance, since loss of Ago2 leads to decreased miRNAs expression and the ectopic presence of argonaute proteins (Ago1–Ago3), as well as of TRBP, give rise to increased levels of pre-miRNA precursor [43]. On the other hand, depletion of argonaute proteins impaired the processing of precursor, indicating the requirement of these proteins for the stabilization of miRNAs [14]. RNA editing of primary transcripts by ADAR (adenosine deaminases acting on RNA) or the uridylation of miRNA 3′-ends could enhance or inhibit the Drosha-DGCR8 and Dicer-TRBP miRNAs processing steps [44–46]. Edited miRNAs can change the miRNA complementarity to target sequences and increase the diversity of miRNAs in the cell [24, 46]. Other modifications on the miRNAs sequences that could promote its stabilization or degradation include the addition of methyl groups to the 3′-ends of miRNAs, the 3′-adenylation, the family of exonucleases that catalyze 3′-to-5′ decay of single-stranded miRNAs, and the 5′-to-3′ exoribonuclease 2 (XRN-2) [14]. Nevertheless, the mechanisms that control the miRNA turnover are not yet completely identified.

microRNAs are present in blood platelets, erythrocytes, and leucocytes. They are unusually stable in plasma and resistant to some hard conditions such as boiling, low and high pH, and long storage and can withstand repetitive freezing and thawing cycles [47–50]. They are also found in saliva, urine, tears, and breast milk and in other body fluids. Circulating miRNAs are sheltered from endogenous RNAse activity [47] since they are linked to lipid-based carriers in a form associated or nonassociated to vesicles. The nonvesicle forms account for 80% to 90% of total circulating microRNAs, which are bonded to RNA-binding proteins in very stable complexes, like argonaute 2 (Ago2) and nuclephosmine 1 (NPM1), or to lipoproteins complexes, such as high-density lipoproteins (HDL) [49–51]. In the cytoplasm, bioactive miRNAs can also be incorporated into microparticles derived from multivesicular bodies (exosomes and microvesicals), which are then released from the cell and are involved in the transfer of genetic information between cells, acting as mediators of cell-to-cell communication [49, 50, 52]. miRNAs are, therefore, implicated in physiological processes such as the regulation of the immunity and angiogenesis or cellular migration, but are also involved in pathological conditions, such as tumor development [52]. It has also been shown that miRNAs are released by apoptotic cells [53, 54].

Because of their stability in the circulation, miRNAs are currently used as potential biomarkers in a wide range of diseases. They are easily detected in a quantitative way by real-time polymerase chain reaction (qRT-PCR) or microarrays and by other less frequently used identification methods, such as traditional northern blotting, PCR-based restriction fragment length polymorphisms (PCR-RLFP), ligation based measurement, and direct sequencing using next generation sequencing (NGS) platforms [55]. The ideal biomarker must be accessible using noninvasive methods, be sensitive and specific for the disease, allow early detection, be sensitive to pathologic changes, and have a long half-life within the sample and the ability of being rapidly and accurately detected [49, 55, 56].

In pathological conditions, such as cardiovascular diseases or cancer, there are characteristic patterns of circulating miRNAs that can be used for diagnostic and monitoring purposes. Since different features of a disease might result in altered types and amounts of plasma miRNAs, a combination of miRNAs will provide a more sensitive and specific diagnostic. Moreover, they could give insights for the intermediate end points in clinical trials [56].

## 4. Modulation of miRNAs

The understanding of miRNA biogenesis and action on target mRNAs is significantly advanced. Since miRNAs act as “fine tuners” and/or “safeguards” to maintain the homeostasis [28], the development of miRNA-based therapeutics relies on antisense technologies for inhibition or replacement of miRNA activity. Indeed, miRNAs can be modulated by oligonucleotides composed of high-affinity nucleotide mimics, designated miR-mimics, and single-stranded antisense oligonucleotides, termed antimiRs or antagomirs. A potential advantage of miRNAs therapy compared to conventional therapies is that they target multiple genes [27, 57] involved in the same pathway and not a single protein, resulting in treatments of high specificity, once they have precise and distinct gene targets in the defined disease pathway [58]. So far, miRNA therapy to treat cardiovascular disorders in human clinical trials is still in the preclinical phase. The antagomir, miravirsen, an anti-miR-122, has been effectively tested in a phase-II trial for the treatment of patients with hepatitis C virus infection [59]. The efficacy of miRNA-based therapies in preclinical small- and large-animal models provides an important tool for the clinical use and development of such treatments for cardiovascular disorders in the near future [60].

The miR-mimics are used when there is a downregulation of a miRNA, as a consequence of a disease, with the aim of increasing a specific miRNA level. Mimics are artificial small nucleotide sequences, similar to pre-miRNAS, which are recognized by the miRNA biogenesis machinery or loaded into the RISC, acting as the endogenous miRNA of interest and blocking gene expression [58, 61–63]. They can be incorporated into a lipid-based formulation improving their stability and cellular uptake. However, since the miR-mimics must act only on the tissue targets, the lipid-based preparations have the disadvantage of increasing the cellular uptake outside of the target organ or tissue. This can be overcome with the use of viral vectors that directly deliver microRNAs [58, 61, 63]. Additionally, successful delivery of oligonucleotides is done by injection since they are soluble in water or normal saline; no oral formulation has been developed [60, 64]. miR-mimics provide an attractive strategy to modulate myocardial miRNAs that are downregulated.

Antagomirs are used when pathological conditions result from the upregulation of a specific miRNA. Antagomirs are modified antisense oligonucleotides (morpholinos) artificially synthesized that thoroughly target the mature miRNA sequence, thereby increasing the amount of mRNA target of that specific miRNA [61, 62, 65]. Antagomirs sequences can be modified in order to improve their stability, binding efficacy, and cellular uptake. These modifications include the use of 2′-*O*-methyl (OMe) and 2′-*O*-Methoxyethyl (MOE) modified oligonucleotides, antisense-based oligonucleotides (ASOs), modified synthetic anti-miRNA oligonucleotides (AMOs), locked nucleic acid (LNA) oligonucleotides, single-stranded antimiRs, cholesterol conjugated antagomirs, liposomes, and nanoparticles [58, 61, 62, 65]. The antimiR can exert its function at different levels of miRNA biogenesis by interfering with pri-miRNAS and with the export of pre-miRNA, preventing its processing or access into the RISC, acting as a competitive inhibitor, linking the mature miRNA within the RISC or hybridizing with the specific miR target, preventing the binding of the miRNA to the respective mRNA [58].

Another way of inhibiting miRNA function is achieved with sponges, masking, and erasers. Sponges prevent the binding of the miRNA to its target miRNA. They contain, in their 3′-UTR, multiple tandem binding sites to the miRNA of interest, with perfect or imperfect complementarity, and act as competitive inhibitors, blocking a whole family of related miRNAs that have the same target site [58, 61–63]. Masking uses oligonucleotides that modulate a specific miRNA target, since they display a perfect complementarity to the target miR [58, 63]. Erasers are also oligonucleotides with tandem repeats of a specific sequence of the complementary antisense sequence of the miRNA [58, 62, 63]. Both, masking and erasers, prevent the miRNA function.

The possible routes of* in vivo* application of miRNA antagonists or mimics to target several cardiovascular cell types are the administration of intravenous, retroorbital, subcutaneous, inhalative, intraperitoneal, transcoronary, or intramuscular injections, but the best method of injection to target cardiovascular tissues is still unresolved. In a few cardiovascular models, site-specific delivery of miRNAs was achieved by cardiac or intracoronary injection [60, 64].

## 5. microRNAs in Age-Related Cardiovascular Diseases

Recent evidence have shown circulating miRNAs as novel biomarkers for cardiovascular diseases since their concentrations in serum are stable and can be reproducibly detected among healthy individuals and their levels are altered in a variety of clinical conditions [66].

Cardiovascular diseases include several pathological disorders, namely, the coronary artery disease (CAD) and its major complication, the acute myocardial infarction (AMI), heart failure (HF), diabetes mellitus, stroke, essential hypertension, and acute pulmonary embolism [66]. The heart suffers complex changes during ageing that include hypertrophy, altered left ventricular diastolic function, reduced left ventricular systolic reverse capacity, increased arterial rigidity, and impaired endothelial function [5, 67, 68]. With age, the apoptotic and necrotic processes lead to a decrease of cardiomyocytes, an increase of oxidative stress promoting a proinflammatory and profibrotic environment, an impaired neovascularization capacity due to a reduction of proangiogenic functions, and a decrease capacity of progenitor cells of the bone marrow-derived cell to contribute to functional repair [5, 69, 70].

Considering the essential role of miRNAs in the mediation of biological events, any change in homeostasis leads to an alteration in miRNA expression profile [57, 71]. Overexpression, deletions, epigenetic modifications, or single-nucleotide polymorphisms in the mature miRNA may result in elimination or variations in the binding affinity to the target mRNA, triggering gene regulation imbalances in both normal and disease conditions [57]. Moreover, a single mRNA may be targeted by several miRNAs and a single miRNA can target many genes [27, 57]. The studies on the epigenetic regulation of CVD-related pathways have been growing with the goal to define novel and potential useful disease markers, like the circulating miRNAs which are tissue-cell specific [49, 72]. Actually, some plasma miRNAs are quite particular of cardiovascular pathologies and, thus, may be used for diagnostic, monitoring purposes, and clinical trials [49, 72].

### 5.1. Coronary Artery Disease

CAD is caused by the formation of atherosclerotic plaques and may progress to myocardial ischemia, as a consequence of thrombosis or of acute coronary atherosclerosis [49, 72]. All cellular components required for plaque or thrombus formation, in response to endothelium activation or damage, may potentially release microRNAs in circulation or, eventually, remove miRNAs modifying the levels of circulating miRNAs. Thus, the evaluation of miRNAs might be used to identify patients at risk of CAD [56, 72].

The relative expression level of 129 microRNAs was analyzed in the peripheral blood mononuclear cells (PBMCs) of CAD patients suffering from either stable angina pectoris (SAP) or unstable angina pectoris (UAP) [73]. An increase in miR-135a and a decrease in miR-147 levels were observed in CAD patients compared to healthy controls. Patients with UAP showed a highly significant upregulation of miR-134, miR-370, and miR-198 compared to those with SAP, and this may predict the clinical outcome of the disease. In addition, the elevated expression of miR-370 can possibly be used to stratify patients that are at risk for acute coronary events from those with stable CAD. These findings suggest that CAD patients exhibit a microRNA-driven change in the inflammatory capacity of blood mononuclear cells [73]. Five miRNAs (miR-19, miR-21, miR-146, miR-155, and miR-133), identified in microparticles (MPs) from plasma, were elevated in patients with acute coronary syndrome (ACS), when compared with patients with CAD [74]. In the absence of myocardial necrosis leading to increased plasmatic levels of the cardiac-specific protein Troponin; the diagnosis of stable angina (SA) or unstable angina (UA) using specific miRNAs showed that miR-1, -122, -126, -133a, -133b, and miR-199a were positively regulated in both UA and SA patients, while miR-337-5p and miR-145 exhibited an upregulation only in SA or UA patients, respectively, compared to controls [75]. A positive modulation of miR-1, miR-126, and miR-485-3p characterized SA patients versus controls. The cluster of miR-1, miR-133a, and miR-126 correctly classified UA patients compared to controls. However, a set of miRNAs that allowed UA to be distinguished from SA was not found [68]. A distinct miRNA profile of upregulation for miR-106b/25 cluster, miR-17/92a cluster, miR-21/590-5p family, miR-126*, and miR-451 was observed in the plasma of patients with typical unstable angina and angiographically documented CAD, as compared to individuals with noncardiac chest pain (control group). microRNA expression in MPs form and in plasma showed also increased levels for miR-106b, miR-25, miR-92a, miR-21, miR-590-5p, miR-126*, and miR-451, indicating that MPs may contribute to the deregulation of circulating miRNA expression in vulnerable CAD patients [69]. Besides, activated platelets play a critical role in the pathophysiology of CAD, may have a mechanism to selectively transport miRNAs into MP, and are a source of miRNAs [74, 76].

In premature CAD patients, microarray analysis of the relative expression levels of platelet miRNAs showed an upregulation of six miRNAs (miR-340*, -451, -454*, -545 : 9.1, -615-5p, and -624*) and a downregulation of miR-1280, compared to controls. The upregulation of miR-340* and miR-624* was validated by qRT-PCR, in a larger cohort, and observed in both young premature CAD and in members of families with premature CAD [77].

Circulating levels of the muscle-enriched miR-499 (20-fold), miR-133a (11-fold), and miR-208a (5-fold) were significantly elevated in the aorta of troponin-positive acute coronary syndrome patients, compared with patients with CAD [78]. Indeed, miR-499 concentration gradients were significantly correlated with the extent of myocardial damage, as measured by high-sensitivity cardiac troponin T (hs-cTnT). In contrast to the muscle-enriched miRs, the endothelial cell-enriched miR-126 demonstrated an inverse transcoronary concentration gradient, suggesting either uptake or degradation of miR-126 during passage through the coronary circulation in patients with troponin-positive ACS. This different regulation of circulating miRs during the transcoronary passage might provide important insights to determine their role as cardiac biomarkers [78]. MiR-499 is implicated in transcriptional and posttranslational regulation of pathological hypertrophy [79]. MiR-126 directly represses the targets' sprouty-related EVH1 domain-containing protein 1 (SPRED1), vascular cell adhesion molecule 1 (VCAM1), and phosphatidylinositol 3-kinase (PIK3) regulatory subunit beta R2/p85-beta (R2/p85-beta). SPRED1 and PIK3R2 negatively regulate vascular endothelial growth factor (VEGF) signaling by the mitogen-activated protein (MAP) kinase and PI3K pathways, respectively. Thus, miR-126, in addition to directly targeting VEGS, promotes VEGF signaling, angiogenesis, and vascular integrity by inhibiting protein production of endogenous VEGF repressors within endothelial cells [80]. The effect of genetic variations in miRNAs evaluated in a Chinese population of 295 CAD patients, by PCR-RLFP, did not reveal any association of the two SNPs in miR-196a2 (rs11614913) and miR-499 (rs3746444) with the risk of CAD incidence [81]. The evidence that miR-146a (rs2910164) polymorphism was related with an increased risk and susceptibility of CAD, in individuals carrying GC and CC genotype, was evaluated in two CAD groups, a Chinese Han population [81] and in young South African Indian patients [82].

Reduction of inflammation-associated miR-155 and smooth muscle cell-associated miR-145 in the whole blood of CAD patients was consistent with the reduced levels found in plasma [72, 83, 84]. This alteration of circulating levels of miR-155 indicates that its downregulation is not limited to endothelial or vascular miRNAs [83].

MiR-126, miR-130a, miR-221, miR-222, and miR-92a were consistently reported as highly expressed in endothelial cells (ECs). Using miRNAs arrays for serum or plasma of controls and CAD patients, Fichtlscherer and coworkers reported that miR-126, miR-92a, and miR-17, predominantly expressed by endothelial cells, were downregulated, while cardiac muscle-enriched miRNAs (miR-133 and miR-208a) were overexpressed though without statistical significance [83]. Another study in CAD patients showed that the expression of miR-126 was decreased, while miR-221, miR-222, and miR-92a were enhanced, and miR-130a remained at the same level as the controls [85]. MiR-221 and miR-222 are known to indirectly repress endothelial nitric oxide synthase required for endothelial progenitor cell function. Thus, the increased expression of these miRs in patient-derived cells may contribute to the well-established reduced number and functional activity of endothelial progenitor cells in patients with CAD [86].

Patients with peripheral arterial disease, specifically atherosclerosis obliterans, presented an increase in serums miR-21, miR-130a, miR-27b, and miR-210, whereas miR-221 and miR-222 were decreased [87]. The increased levels of miR-130a and miR-27b were correlated with disease severity [87]. However, the potential function of these two miRs in atherosclerosis obliterans should be further analyzed.

The pattern of expression of certain miRNAs can be modified when patients are subjected to medication. It was reported that levels of miR-146a/b were higher in peripheral blood mononucleated cells (PBMCs) of CAD patients when compared with non-CAD patients, but treatment with angiotensin II receptor blocker inhibitors and statins reduced monocytic levels of miR-146a/b [88]. This study also suggested that dysregulation of miR-146a/b expression may contribute to prolonging the activation of Toll-like receptor 4 (TLR4) which, in turn, may induce miR-146a/b expression as a negative regulator and, so, induce progression of coronary atherosclerosis in patients with CAD [88]. In another study, the levels of miR-221 and miR-222 were significantly higher in CAD patients compared to the non-CAD group. Although, after lipid lowering therapy (LLT) with atorvastatin, CAD patients showed an increase in the number of endothelial progenitor cells (EPCs) and a decrease in miR-221/222 levels [86]. In the whole blood, miR-140-3p and miR-182 were enhanced in CAD patients [89], and miR-19a, miR-484, miR-155, miR-222, miR-145, miR-29a, miR-378, miR-342, miR-181d, miR-150, and miR-30e-5p were reduced [84]. However, some of these miRNAs (miR-19a, miR-145, miR-155, miR-222, miR-342, miR-378, and miR-30e-5p) were significantly reduced in patients treated with angiotensin-converting enzyme (ACE), indicating that medication may alter miRNA expression [84]. In another study, it was shown that atorvastatin treatment did not affect miR-126 and miR-130a, but enhanced the production of miR-221, miR-222, and miR-92a in cultured EPCs from patients with CAD. The difference between the two studies, from Minami et al. and Zang et al., might be explained by the method of atorvastatin administration, which in the latter work was directly given to cultured cells [85].

The studies done on the circulating miRNAs as diagnostic makers for CAD are summarized in Table 1.

**Figure 2:**
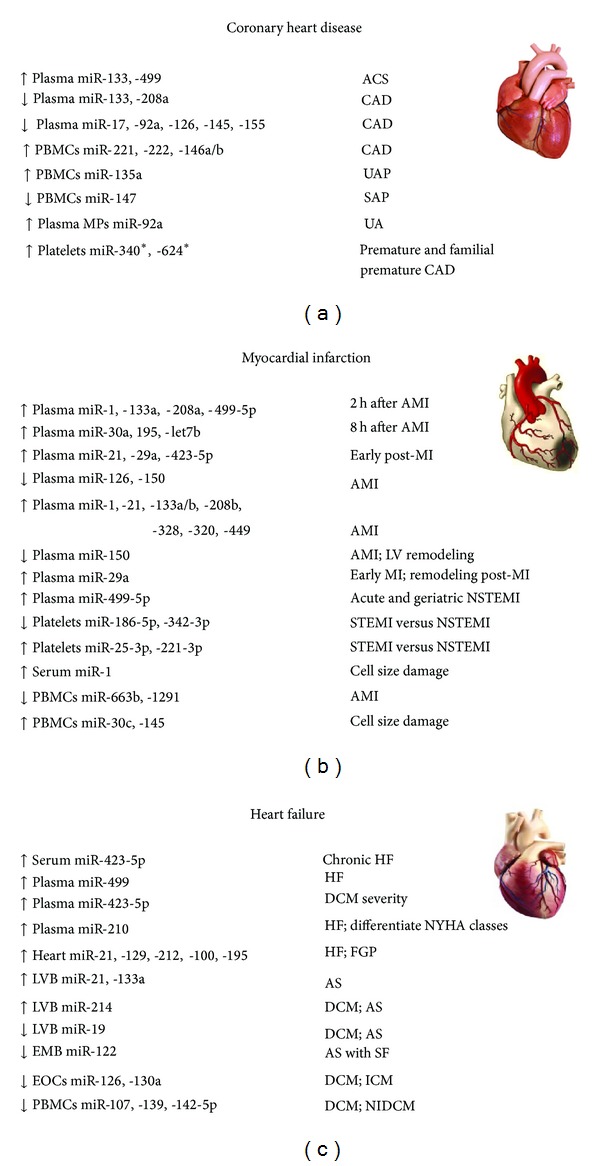
The most relevant miRNAs reported as promising biomarkers in coronary heart disease (a), myocardial infarction (b), and heart failure (c). ACS: acute coronary syndrome; AMI: acute myocardial infarction; CAD: coronary heart disease; DCM: dilated cardiomyopathy; Down: downregulated; EMB: endomyocardial biopsy; EOCs: early outgrowth cells; FGP: fetal gene program; h: hours; HF: heart failure ICM: ischemic cardiomyopathy; LV: left ventricle; LVB: left ventricle biopsies; MI: myocardial infarction; MPs: microparticles; NYHA: New York Heart Association; NSTEMI: non-ST elevation myocardial infarction; PBMCs: peripheral blood mononuclear cells; SAP: stable angina pectoris; SF: severe fibrosis; STEMI: ST elevation myocardial infarction; UA: unstable angina; UAP: unstable angina pectoris; UP: upregulated.

In conclusion, analysis of miRNAs profiles released into the blood stream from cells and tissues of the cardiovascular system in CAD patients revealed the downregulation of miR-17, miR-92a, miR-126, miR-145, and miR-155 and the upregulation of miR-133 and miR-208a. Some miRNAs allowed the identification of patients with elevated risk of developing acute coronary syndrome (miR-133a and miR-499), and others (miR-135a and mir-147) discriminate between patients with stable and unstable angina pectoris. Several of these patterns could change with the expression of miRNAs from microparticles (miR-92a), in patients with unstable angina, or from platelets (miR-340 and miR-624) as shown in young patients with premature development of CAD (Figure 2(a)). The identification of RNA signatures and its SNPs, in patients with CAD, is crucial for the diagnosis, stratification, treatment, and correlation with clinical parameters of this heart pathology.

### 5.2. Myocardial Infarction

Myocardial infarction (MI) or acute myocardial infarction (AMI) is usually known as a heart attack. Chest pain is the most common symptom of AMI, and when due to ischemia of the heart muscle, it is termed angina pectoris.Acute coronary syndrome (ACS), characterized with chest pain, refers to any group of symptoms attributed to obstruction of the coronary arteries. ACS frequently occurs as a result of ST elevation myocardial infarction (STEMI), non-ST elevation myocardial infarction (NSTEMI), or unstable angina [90].

Several studies have been performed to establish the miRNA profile associated with AMI and MI. miR-1 is the most abundant heart and muscle-specific miRNA. Under physiological conditions, only a small amount of miR-1 is released into the blood. After damage, the released amount of miR-1 was associated with the extent and size of cardiac cell injury. The levels of circulating cell-free miR-1 were significantly increased in patients with AMI and had a positive correlation with serum creatine kinase-MB (CK-MB) levels [91]. Moreover, in rats with acute myocardial infarction the serum miR-1 concentration had a strong positive correlation with myocardial infarct size [91]. These outcomes proposed serum miR-1 as a novel sensitive diagnostic biomarker for AMI [91–93]. miR-1 controls cardiomyocyte growth responses by negatively regulating the calcium signaling components calmodulin (CaM), myocyte-specific enhancer factor 2A (MEF2A), and the transcription factor GATA-4 [94]. It was shown that plasma levels of miR-1 were upregulated and miR-126 were downregulated, with a high sensitivity and specificity for the detection of AMI patients after the onset of symptoms (from 4 hours to 1 week) compared to controls. Furthermore, miR-1 and mir-126 and cardiac troponin-I (cTnI) expression presented the same trend [93]. Modulation of the expression of miR-126 in endothelial cells* in vitro*, with a morpholino antisense to miR-126, and* in vivo*, using a transgenic (Tg) zebrafish, showed that miR-126 directly repress negative regulators of the VEGF pathway, including the SPRED1 and PIK3R2/p85-*β* [80]. The reduced expression of miR-1915 and upregulation of miR-181c* was suggested as a hallmark of the very early phase of myocardial infarction [95].

miR-1, miR-133a, and miR-499 are expressed in skeletal muscle, and the increased level of these miRNAs in plasma might be due to skeletal muscle damage. These three miRNAs presented high levels in plasma from the AMI patients [96]. Other reports also demonstrated increased levels of cardiac-enriched miR-499 in patients with MI and AMI [97–99], as well as in hs-cTnT [76, 97, 99] and CK [99], thus providing an accurate diagnosis of MI. MiR-499 was increased in human, in murine cardiac hypertrophy and cardiomyopathy, by acting, directly or indirectly, on the expression of cardiac protein kinases and phosphatases. Whole-genome RISC sequencing analysis identified 67 direct miR-499 target mRNAs that included the AKT and MAP kinase pathways, and the phosphatases tensin homolog (PTEN) and the PH domain leucine-rich repeat protein phosphatase (PHLPP1) [79].

The miR-208 family includes two subfamilies: miR-208a and miR-208b. An upregulation of miR-208 was observed in samples of infarcted heart tissue from patients with MI compared to healthy adult hearts [100]. The concentration of miR-208, produced in the heart, was increased in the plasma of rats after isoproterenol-induced myocardial injury but not in rats with hypertensive cardiac hypertrophy, suggesting that the hypertrophy was not enough for miR-208 to leak out of cardiac myocytes [101]. However, transgenic overexpression of miR-208a in the heart was sufficient to induce hypertrophic growth and arrhythmias in mice. On the contrary, the lack of miR-208a in a transgenic mouse did not affect the viability or cause gross morphological heart defects. Electrocardiogram analysis of miR-208a in transgenic* Mir208a* ^−/−^ mice demonstrated that miR-208a was required for the proper cardiac conduction and expression of the cardiac transcription factors, like homeodomain-only protein (HOP) and GATA4, and for the gap junction protein connexin 40 [102]. MiR-208a was undetectable in plasma from healthy people, but was upregulated in plasma from AMI patients. In addition, it has a higher sensitivity in early setting of AMI, especially within 4 h of the onset of symptoms, making miR-208a a more reliable biomarker for AMI diagnosis in both, human and rats [103]. In a pig reperfusion model, miR-1, miR-133a, and miR-208b increased rapidly in plasma with a peak at 120 min, while miR-499-5p remained elevated for longer periods [104]. Both miR-208a and miR-208b target thyroid hormone receptor associated protein 1 (THRAP1) and myostatin, which are negative regulators of muscle growth and hypertrophy [102].

In acute MI patients, miR-1, miR-133a and miR-208a increased rapidly and peaked within 2 hours following the onset of cardiac ischemia, whereas miR-499-5p continued to increase even after 2.5 hours [96, 104]. Moreover, miR-208b exhibited the highest increase and these values were correlated with troponin [104]. The association of miR-208b and AMI was also observed in a cohort of 444 acute coronary syndrome (ACS) patients [105] and in a cohort of 510 patients with MI [99]. The indirect evidence that miR-208b and miR-499-5p were released specifically from the human heart was obtained by performing a study in four samples from the coronary sinus, before and after cardioplegia and reperfusion in patients undergoing coronary artery bypass grafting (CABG): miR-208b and miR-499-5p emerged in the coronary sinus immediately after, but not before, cardioplegia, supporting the suggestion that these miRNAs were in fact released directly from the myocardium following tissue damage [106]. The increased levels of these cardio-enriched miRNAs in the blood of MI patients were related to a reduced systolic function after MI and with the risk of death or heart failure [106]. microRNA-133a concentrations were determined in 216 patients with STEMI undergoing primary angioplasty less than 12 hours after symptom onset of AMI and increasing levels of circulating miR-133a were associated with decreased myocardial salvage, larger infarcts, and more pronounced reperfusion injury [107].

Cheng and Zhang found that miR-21 expression was significantly downregulated in infarcted rat heart areas, but was upregulated in the border areas, at 6 h and 24 h after AMI [108]. This was previously observed in another animal study using cultures of rat myocytes* in vitro*, suggesting an important role for miR-21 in the early phase of MI [109]. In addition, it was demonstrated, in an* in vivo* cardiac ischemic preconditioning (IP) model, that miR-21 was quickly upregulated by IP, an effect that was inhibited by knockdown of cardiac miR-21 expression [110].* In vivo*, the protective effect of miR-21 on ischemic injury was confirmed in a cardiac cell ischemic/reperfusion (H/R) model [110], by reducing cardiac cell apoptosis via its target, the programmed cell death 4 (PDCD4) [109, 110].

Plasma levels of certain microRNAs, such as miR-21 and miR-29a, increase early post-MI, whereas others, such as miR-1, miR-133a, and miR-208, remained persistently elevated up to 3 months post-MI. Furthermore, higher levels of miR-29a early post-MI were related to greater remodeling after post-MI [111]. In humans and mice with AMI, plasma levels of miR-1, miR-133a, miR-133b, and miR-499-5p were upregulated. Increased levels of miR-1 and miR-133a were detected early just after chest pain and before elevation of CK and cTnT [112]. In the same study, using a mouse myocardial infarction model (C57BL/6 male mice) at 24 hours after coronary ligation, the expression levels of cardiomyocyte-specific miRNAs, such as miR-1, miR-133a, miR-208, and miR-499, were reduced in the infarcted region. However,* in situ* hybridization of miR-133a clearly showed the absence of miR-133a in cardiomyocytes, suggesting miR-133a as a marker for cardiomyocyte death [112]. In contradiction, there is a study that found a downregulation of miR-1 and miR-133a/b in infarcted heart tissue patients compared to healthy adult hearts [100].

The temporal changes of miR-133a and miR-423-5p were measured in the plasma of post-MI patients, for one year, and they were not considered as predictors of left ventricular function and remodeling, despite their increased concentrations [113]. However, mir-150 was identified as biomarker of left ventricular remodeling [114]. The circulating levels of miR-133a can be used as a predictor for diagnosing AMI and coronary heart disease (CHD), since its levels increased in a time-dependent manner in the early phase of AMI and were positively correlated with cTnT in AMI patients, associating miR-133a with the occurrence and severity of coronary atherosclerosis in CHD patients [115].

The expression of miR-423-5p in plasma significantly increased at the beginning of an AMI event, and this miR has been suggested as a potential early marker of myocardial necrosis [116]. Another study demonstrated the increased levels (3–10 fold) of miR-1, miR-21, miR-133a, miR-423-5p, and miR-499-5p in the plasma of geriatric patients with acute non-ST elevation myocardial infarction (NSTEMI). The authors proposed circulating miR-499-5p as a sensitive biomarker of acute NSTEMI, in a geriatric population, while miR-21 was described as upregulated in patients with acute MI [117]. Increased circulating levels of miR-1, miR-21, miR-133a, and miR-499-5p were previously reported in patients and animal models with AMI [76, 91, 103]. MiR-423-5p was also found elevated in the circulation of patients with CHF [118].

Other reports revealed a miRNA profile with an upregulation of miR-25-3p, miR-221-3p, and miR-374b-5p in STEMI patients compared to NSTEMI; miRNA-30d-5p expression was associated with plasma, platelets, and leukocytes in both STEMI and NSTEMI patients; miRNAs (221-3p and 483-5p) were correlated with plasma and platelets only in NSTEMI patients [119]. Expression of plasma miR-92a-3p and miR-30d-5p, platelet miR-186-5p and miR-342-3p, and PBMCs miR-374b-5p was significantly lower in patients with STEMI as compared with NSTEMI. In contrast, plasma miR-25-3p and miR-374b-5p, platelet miR-25-3p and miR-221-3p, and PBMCsmiR-25-3p and miR-221-3p were significantly higher in patients with STEMI than in NSTEMI [119]. Circulating miR-133 or miR-328 were upregulated in plasma and whole blood, and were correlated with the myocardial damage marker, cardiac troponin I, indicating that these two miRNAs may be released from damaged myocardium into circulating blood when heart injury occurs [120]. The levels of miR-223, miR-320a, and miR-451 were higher than the cardiomyocyte-enriched miR-133a, miR-208b, and miR-499, but the circulating levels of miR-208b, miR-320a, and miR-499 were significantly higher in patients with AMI and in patients with STEMI compared to NSTEMI [121]. Mir-208b was exclusively released by myocardial injury and not by muscle injury, as occurred for miR-133a and miR-499 [72], demonstrating its highest diagnostic accuracy for AMI [120]. These six miRNAs studied had no or very low predictive value for the occurrence of future AMI or death during long-term follow-up (730 days) [121].

In patients who survived AMI, miR-155 and miR-380 were differentially expressed and considerably higher in patients who subsequently died due to cardiac causes [122]. MiR-133a and miR-208b were significantly associated with the risk of death in patients with acute coronary syndromes [105]. Meder and collaborators identified 121 miRNAs, which were significantly deregulated in AMI patients. Among these, miR-1291 and miR-663b showed the highest sensitivity and specificity, and miR-30c and miR-145 levels were correlated with infarct sizes as estimated by troponin T release [123]. Circulating miR-30a, miR-195, and let-7b were proposed as biomarkers for AMI, as their levels reached a peak at 8 h after AMI and were well correlated with the plasmatic concentrations of cTnI [124]. More recently, the miR-17-92 cluster was identified as key regulator of cardiomyocyte proliferation in embryonic, postnatal, and adult heart in transgenic and knock-out mice, by targeting PTEN [125].

The studies performed for the recognition of circulating miRNAs as diagnostic makers for MI are concise in Table 2.

It is clear that miRs are responsible for the regulation of several biological functions, and the alterations in cardiovascular diseases have been analyzed through the identification and evaluation of miRNAs levels and profiles in patients as in animal models. For acute myocardial infarction, there are muscle cells-enriched miRNAs (miR-1, -21 miR-133a/b, miR-499, miR-663b, and miR-1291), cardiac specific microRNAs (miR-208), and smooth muscle-enriched microRNAs (miR-30c and miR-145) that displayed good associations to troponins. Mir-499 and miR-208b upregulation was also correlated with the hs-cTnT and CK, making these miRNAs useful tools for MI and AMI diagnosis. A rise of miR-499 was also found in HF patients. The elevated levels of miR-499-5p suggest its use as a biomarker for acute non-ST elevation myocardial infarction, in geriatric patients. In line, patients with STEMI and NSTEMI can be differentiated according to specific microRNAs profiles expressed in plasma, leucocytes, and/or platelets. Several microRNAs, like miR-29a or miR-150, are involved in cardiac remodeling after MI (Figure 2(b)). Overall, these studies indicate the usefulness of blood miRNAs as stable biomarkers for myocardial infarction and related conditions. microRNA induction or repression after myocardial infarction triggers downstream events in a cell-type-specific manner, and interference with endogenous miR expression might regulate overall cardiac function.

### 5.3. Heart Failure

Heart failure is a leading cause of death in industrialized nations especially in the aging population. Heart failure, often called congestive heart failure, occurs when the heart is unable to provide sufficient pump action to maintain blood flow to meet the body needs. End-stage heart failure is featured by significantly disturbed neurohormonal and mechanical stimuli to the heart. Dilated cardiomyopathy (DCM) is characterized clinically by left ventricular dilatation, ventricular wall thinning, and homogeneous myocardial dysfunction leading to congestive heart failure [126] and right ventricular dysfunction [127]. The main common cause of systolic heart failure, particularly in developed nations, is ischemic cardiomyopathy (ICM), resulting from significant coronary artery disease as a consequence of multiple factors, including progressive heart failure and tachyarrhythmia [128]. Aortic stenosis (AS) is the most usual form of valvular heart disease and is frequently caused by left ventricular outflow tract obstruction in adults [129]. In an attempt to find diagnostic markers for these different etiologies, studies in humans and animal models have contributed for the identification of genome wide-expression miRNA profiles in heart failure, which is specific for each individual form of heart diseases [11].

microRNA expression patterns were very similar between patients at end-stage heart failure and fetal human heart tissues, showing an upregulation of miR-21, miR-129, and miR-212. Moreover, transfection of isolated adult rat cardiomyocytes with this set of fetal miRNAs (miR-21, miR-129, and miR-212) induced morphological changes in neonatal cardiomyocyte, causing hypertrophy and activation of fetal gene program [130]. In idiopathic end-stage failing human hearts, northern blot analysis of the hypertrophy-regulated miRNAs revealed increased expression of miR-24, miR-125b, miR-195, miR-199a, and miR-214, whereas the expression for miR-23 appeared to be variable within the nonfailing and failing groups. The increased level of these miRNAs (miR-21, -23, -24, -125b, -195, -199a, and -214) was confirmed by northern blot analysis of cardiac RNA from wild type littermates and CnA Tg mice expressing activated calcineurin A (CnA) in the heart [131]. A subset of specific miRNAs was identified in patients with dilated cardiomyopathy and ischemic cardiomyopathy showing that miR-100 and miR-195 were upregulated while miR-92 and miR-133b were downregulated [132]. Myocytes exposed* in vitro* to overexpression of miR-195 promoted cardiac growth and may even override the inhibition of miR-1 on cardiac growth [131]. Cardiac overexpression of miRNA-195 in transgenic mice leads to hypertrophic growth and myocyte disarray, resulting in dilated cardiomyopathy and heart failure [131]. These changes were also observed in primary cultures of neonatal cardiac myocytes using miRNAs mimics or inhibitors of miR-100 and miR-133b, suggesting that both contribute to regulate the fetal gene program [132]. MiR-100 seems to have a specific role in the adult isoforms of cardiac genes and miR-133 appears to regulate the Rho family of small GTP-binding proteins [132].

The upregulation of miR-24, after 4 hours of left anterior descending (LAD) coronary artery occlusion, was detected in primary cultured rat cardiomyocytes exposed to ischemia and in a rat model of myocardial ischemia. Moreover, overexpression of miR-24 mimic sequence and transfection of miR-24 inhibitor, respectively, inhibited and intensified both apoptosis and necrosis in ischemic myocytes, suggesting a possible role of miR-24 in cardioprotection [133]. The protective effects of miR-24 against myocardial ischemia is related to the inhibition of the gene encoding the B-cell leukemia/lymphoma-2 like protein 11 (BCL2L11) in rat cardiomyocytes [133].

In HF of different etiologies (ICM, DCM and AS), miR-214 was upregulated while miR-19 was downregulated in DCM and AS, but not in ICM [10]. A specificity of miRNA expression profile for each form of heart disease was also observed [11]. Mir-214 was upregulated in several disease prototypes of hypertrophy and heart failure [131]. In mice, miR-214 plays a cardioprotective function against excessive Ca^2+^ uptake and cardiomyocyte death* in vivo* and* in vitro* [134]. MiR-214 protected the heart against IR injury by inhibiting its targets: sodium-calcium exchanger 1(NCX1), Ca^2+^/calmodulin-dependent protein kinase II *γ* (CaMKII*γ*), cyclophilin D (CYPD), and BCL2-like 11 (BIM) genes [134].

Data from different studies in patients with heart failure revealed an upregulation of miRNAs (miR-21, -23a, -125b, -195, -199A, -214, and -342) and a downregulation of miRNAs (miR-1, -7, -29b, -30, -133, -150, and -378) [11, 126, 130–132, 135]. Patients with end-stage DCM showed a consistent pattern of eight miRNAs, with a downregulation of miR-7 and miR-378 and an upregulation of miR-214 and miR-181b. These miRNAs were identified for the first time in these patients and in mice (C57Bl/6) that underwent transverse aortic constriction (TAC) surgery [126]. From these eight miRNAs, only a subset of four miRNAs (miR-7, -378, -214, and -181b) was altered in the beginning of cardiac dysfunction at the end-stage heart failure and may potentially deregulate signaling networks, contributing to pathology [126]. The predicted targets for miR-7 are the receptor tyrosine-protein kinase erbB-2 (ERBB2) and collagen1 (COL1) genes, which are nodal molecules; for miR-214 are the hepatoma-derived growth factor (HDGF) and BCL1 genes; and for miR-378 the targets are the cardiotrophin-like cytokine factor 1 (CLCF1) and solute carrier family 2A (SLC2A) genes [126]. In the rat model of myocardial ischemia caused by LAD and in H9c2 cardiomyocytes under the effect of hypoxia, miR-378 expression was downregulated. By preventing caspase-3 expression, the overexpression of miR-378 considerably improved cell viability and inhibited apoptosis and necrosis. The miR-378 inhibitor had opposed effects, indicating the use of this cardioprotector miRNA as a potential therapeutic device for treatment of ischemic heart disease [136].

A search of miRNAs expressed in patients with HF, non-HF forms of dyspnea, and controls identified miR-423-5p and six other miRNAs (miR-18b*, miR-129-5p, miR-1254, miR-675, HS_202.1, and miR-622) with increased circulating levels. MiR-423-5p was upregulated only in subjects with clinical HF. MiR-423-5p and miR-675, but not miR-18b*, were upmodulated in atherosclerotic forms of HF as compared to nonatherosclerotic forms of HF. Analysis of miRNAs that were upregulated in clinical HF and those that were commonly upregulated in subjects non-HF with dyspnea indicated miR-675 as the mostly upregulated in dyspnea [118]. A second group of circulating miRNAs (miR-129-5p, -18b*, HS_202.1, -622, and -1254) was significantly upregulated in non-HF cases, when compared to the healthy controls [118]. A study comparing the profile of circulating microRNAs of patients with heart failure, chronic obstructive pulmonary disease (COPD), other breathless patients, and controls revealed lower levels of expression of miR-103, miR-142-3p, miR-30b, and miR-342-3p in HF patients, but not for miR-423-5p [137], which was previously reported as a diagnostic predictor of HF [118, 130, 138]. Some of these microRNAs have specific targets: the miR-199 family regulates the hypoxia-induced factor-1*α* (HIF-1*α*) and stabilizes the proapoptotic factor p53 [129]; miR-103 is induced in response to hypoxia; miR-27b is involved in pyruvate and lipid metabolism [137]; miR-324-5p is implicated in the hedgehog signaling pathway, increased in angiogenesis, and induced by hypoxia [135, 137].

Elevated serum levels of miR-423-5p, miR-320a, miR-22, and miR-92b, identified in systolic heart failure patients, were correlated with important clinical prognostic parameters, like elevated serum brain natriuretic peptide (BNP) concentrations, a wide QRS, and dilatation of the left ventricle and left atrium [138]. The levels of circulating miR-423-5p were elevated in DCM patients, positively related to N-terminal probrain natriuretic peptide (NT-proBNP) levels and may reflect the severity of DCM [139]. In contrast, another study in patients with a systemic right ventricle and reduced ejection fraction indicated that miR-423-5p levels were not elevated and were similar to controls [133]. This was attributed to the fact that patients were only mildly symptomatic [140] while in the study done by Tijsen and colleagues patients more severely affected were included [118].

Plasma concentrations of miR-126 were reduced in patients with HF, compared to healthy subjects, and inversely correlated with plasma concentrations of BNP, a classic marker of heart failure. Indeed, higher levels of miR-126 were found to be associated with a better clinical condition [141]. Patients with CHF caused by ICM and DCM displayed a pronounced loss of miR-126 and miR-130a in angiogenic early outgrowth cells (EOCs) and in circulating CD34^+^ cells as well as in their respective targets, the SPRED-1 and the homeobox A5 (HOXA5) [142]. These alterations were not found in previous reports [118, 126, 130]. However, experimental studies in miR-126^−/−^ mice suggested a key role for miR-126 in cardiac neovascularization, by modulating angiogenesis* in vivo*. Overexpression of miR-126 prevented the expression of SPRED-1 on the signaling pathways activated by VEGF and fibroblast growth factor (FGF), promoting angiogenesis [143]. Cardiac transplantation angiogenic EOCs from healthy subjects to nude mice stimulated myocardial neovascularization and enhanced cardiac function. However, this effect was not verified after transplantation of angiogenic EOCs from patients with CHF. Transfection of antimiR-126 of angiogenic EOCs from healthy controls diminished the capacity of cardiac neovascularization whereas transfection with miR-126 mimics of angiogenic EOCs from patients with CHF increased cardiac neovascularization and function [142].

Endothelial progenitor cells from patients with ICM and non-ICM expressed several miRNAs differently. Some of them, like miR-107, miR-139, and miR-142-5p, which were previously reported as downregulated in PBMCs of NICM and ICM patients [144], did not show a changed expression in EPCs from patients with ICM and NICM [144]. The pattern of expression of miR-126 (downregulated) and of miR-130a and miR-221 (upregulated) in EPCs from ICM patients as compared to controls [145] was also detected in patients with multiple cardiac risk factors and stable CAD [85]. High levels of miR-508-5p may downregulate its target Glu1, in EPCs, and impair HIF-1*α*, contributing to a reduced survival of patients with CHF caused by NICM [145]. The low expression of miR-126 and the increased expression of miR-508-5p in EPCs were independent prognostic factors associated with an increased risk for cardiovascular death and for a decreased survival [145].

In acute HF, only miR-499 levels were significantly elevated when compared to controls [90]. Although, in another study, the plasma concentrations of miR-499 were below the limit of detection for both, controls and CHF patients [98]. Three miRNAs (miR-107, -139, and -142-5p) were downmodulated in both NIDCM and ICM patients versus control subjects. Other miRNAs were deregulated in only one of the CHF classes analyzed compared with control subjects: miR-142-3p and miR-29b were increased in NIDCM patients, while miR-125b and miR-497 were decreased in ICM patients [144]. In the heart of NIDCM patients, miR-107 and miR-29b were down- and upregulated, respectively [130], and in the ICM class, miR-497 was expressed at significantly lower amounts than in healthy individuals, as also observed in end-stage human failing hearts [18].

The miRNA array analysis of plasma from Dahl salt-sensitive rats with heart failure showed an upregulation of miR-15a, miR-15b, miR-20a, miR-103, miR-130a, miR-130b, miR-195, miR-210, miR-301b, miR-451, and miR-494 [146]. Nevertheless, this array analysis performed in humans with severe heart failure displayed only a significant upmodulation of miR-210 and miR-451 [141, 146]. MiR-451 is greatly expressed in erythroid cells, and the hemolysis commonly observed in patients with congestive heart failure appears to be the cause for the upregulation of this miRNA. The upmodulation of miR-494, that activates the AKT pathway and targets both proapoptotic and antiapoptotic proteins, seems to act as a protecting factor against ischemia/reperfusion-induced cardiac injury [146]. The expression levels of miR-210 in the mononuclear cells of patients with class III and IV of the New York Heart Association (NYHA) were significantly higher than in patients with NYHA II heart failure and healthy controls. Moreover, plasma miR-210 levels were strongly correlated with BNP, a marker of heart failure, making miR-210 a good supplementary prognostic biomarker to evaluate patients with HF [146]. The upregulation of miR-210 and miR-30a was detected in the serum of HF patients and in umbilical cord blood and followed the increased levels of NT-pro-BNP [147]. Expression of miR-210 is induced by HIF-1*α* during hypoxia, and its upregulation was observed in rat H9c2 myocardial cells [146]. In addition, miR-210 might repress iron-sulfur clusters assembly protein (ISCU) that may protect cells from apoptosis [146, 147].

Five miRNAs (miR-101b, -142-3p, -181d, -24-2*, and -450a) were identified as negative regulators of cardiac hypertrophy, since they abrogated the phenylephedrine (PE)-induced hypertrophy in neonatal rat cardiomyocytes, and transfection of antagomirs for these miRNAs significantly exacerbated PE-induced hypertrophic responses [148].

Overexpression of miR-22 in transgenic mice lead to cellular and organ hypertrophic growth compared to the wild type mice, and in cultured neonatal cardiomyocytes miR-22 promoted classic features of hypertrophy [149]. On the other hand, cardiomyocytes transfected with miR-22 mimic induced cardiomyocytes hypertrophy, while miR-22-null mice hearts showed reduced cardiac hypertrophy and cardiac remodeling [150]. MiR-22 inhibited sirtuin 1 (SIRT1) and histone deacetylase 4 (HDAC4), two important epigenetic regulators of cardiac function [150].

MiR-133a is specific for skeletal muscle, promotes myogenic differentiation, and has a critical role in determining cardiomyocyte hypertrophy [151, 152]. In fact, in mice overexpressing miR-133a there was a reduction of hypertrophy by suppression of its cardiomyocyte targets [152] that included RhoA, a GDP-GTP exchange protein regulating cardiac hypertrophy, cell division control protein 42 (Cdc42), a signal transduction kinase implicated in hypertrophy, and the negative elongation factor A/Wolf-Hirschhorn syndrome (NELF-A/WHSC2), a nuclear factor involved in cardiogenesis [151]. Patients with aortic stenosis, who normalized the left ventricular mass (LVM) one year after aortic valve replacement (AVR), exhibited preoperative high levels of circulating miR-133a, compared with patients who presented residual hypertrophy, probably as a consequence of its release by the myocardium into the circulation in the pressure overload condition [153]. As a key regulator of cardiac hypertrophy, miR-133 together with other clinical parameters could be used to predict the potential reversibility of LV hypertrophy after AVR [153].

The myocardial and circulating levels of miR-21 were increased in patients with AS when compared to the group of surgical controls. This overexpression was confined to extracellular matrix, absent in cardiomyocytes, and was inversely correlated with one of its targets, the PDCD4 gene. Therefore, a possible function as a regulator of the fibrotic process that occurs in response to pressure overload in AS patients was suggested for miR-21 [154]. Experiments in human fibroblasts to analyze the myocardial fibrosis in AS patents identified several deregulated microRNAs. The expression of miR-122 was downregulated in patients with severe fibrosis (SF) when compared with non-SF patients and controls, probably through the upregulation of transforming growth factor beta 1 (TGF-b1) [155].

The different studies performed to identify circulating miRNAs as diagnostic makers for MI are succinct in Table 3.

As stated, there are several microRNAs with aberrant expression levels in heart failure. Among them, miR-423-5p was the most closely related to the clinical diagnosis of HF and miR-675 was the most upregulated in dyspnea. The elevated levels of miR-423-5p seem to be associated with the severity of DCM and were positively correlated with BNP concentrations. Experimental studies suggested that miR-126 stimulated cardiac neovascularization and improved cardiac function. MiR-133 levels, together with other clinical parameters, were proposed as a therapeutic approach to predict the regression potential of LV hypertrophy after AVR. The expression of miR-210 allowed distinguishing the extent of heart failure according to the NYHA functional classification. MiR-21 was upregulated in AS patients compared to the surgical controls, while miR-122 was downregulated in AS patients with severe fibrosis (Figure 2(c)). In this way, the assessment of miRNA profiles as a prognostic tool is promising and represents a challenge for the development of miRNA-based therapies for cardiovascular diseases.

## 6. Future Perspectives

The expression of miRNAs is temporally and spatially regulated, and alterations of their physiological expression patterns are associated with several human cardiovascular diseases, suggesting that they may play a role as a novel class of biomarkers or treatment targets for cardiovascular diseases [137]. The expression of miRNAs within myocardium is the most exciting and innovative mechanism in the regulation of myocardial infarction, heart failure, and hypertrophy [11, 126, 130].

Analysis of differently expressed miRNAs, using recent development of sequencing technologies and computational prediction algorithmic methods, revealed that the expected targets are associated with the network of cardiovascular development and function [62, 63]. The evaluation of deregulated miRNAs in distinct disease conditions may help the diagnosis and prognosis, as well as monitor the development of drug targeting and the normalization of miRNAs levels. In that sense, the severity of the affected patients, the different pathophysiologies of systemic right ventricular and left ventricular heart failures, the analysis of larger cohort of patients in each pathology, the time of sample collection, the way miRs are released in plasma by various cells and tissues or different blood components, the medication, and SNPs are important causes to consider for potential setting of disease markers. Aging, senescence, and other associated diseases, like diabetes mellitus, viral infections, immune reactions, stroke, or hypertension, contribute to a different miRNA expression profile but, at the same time, allow the identification of specific miRs for different clinical conditions. In addition, genetic and epigenetic regulations have a primordial role in the microRNAs and gene expression interactions [1–3]. Moreover, it is essential to determine the regulation of signaling pathways by miRNAs, since it will provide an understanding on the global scale of regulation by miRNAs, instead of looking only to individual targets [126]. Currently, modulation of miRs can be achieved,* in vitro* and* in vivo*, by miRNA therapies which are based in the use of antagomirs and miR-mimics.

In conclusion, it is well established that miRNAs are deregulated in most human diseases. The main advantage of miRNA therapeutics is their innovative mode of action, apparently without adverse effects [59]. Currently, the miR delivering system is systemic and not specific to an organ or tissue and consequently has the ability of exerting adverse side effects. It would be interesting to identify circulating miRNAs as markers for diagnosis and prognosis and those that could predict a response to a particular therapy in cardiovascular diseases. However, the development of effective delivery systems, the validation of clinically relevant animal models, and additional medical trials are required to improve our understanding of the molecular mechanisms underlying some diseases, in particular the heart pathologies and the following of degenerative and regenerative processes.

## Figures and Tables

**Table 1:** Circulating miRNAs as diagnostic markers for CAD.

miRNAs	Regulation	Groups	Source	Methods	Reference
miR-126, -17, -92a, -145, -155	Down	Human/36 CAD,	Plasma or	Array	[83]
miR-133, -208a	Up	17 controls	Serum		
					
miR-92a, -126, -133a,	Up ACS versus CAD	Human/31 CAD, 19 ACS;	Plasma	qRT-PCR	[78]
-208a, -499		7 controls			
					
miR-140-3p, miR-182	Up	Human/12 CAD, 12 controls	Whole blood	Array	[89]
					
miR-19a, -484, -155, -222,	Down	Human/10 CAD, 15 controls	Whole blood	Array	[84]
miR-29a, -378, -342, -181d,				qRT-PCR	
miR, 145, -150, -30e-5p					
					
miR-221, -222	Up	Human/44 CAD, 22 non-CAD	PBMCs	qRT-PCR	[86]
					
miR-146a/b	Up	Human/66 CAD, 33 non-CAD	PBMCs	qRT-PCR	[88]
					
miR-19, -21, -146, -155, -133	Up ACS versus CAD	Human/5 CAD, 5 non-CAD	Plasma MPs	qRT-PCR	[74]
					
miR-135a	Up versus controls	Human/25 UAP;	PBMCs	Array	[73]
miR-147	Down versus controls	25 stable SAP; 20 controls			
miR-134, -198, -370	Up UAP versus SAP				
					
miR-1, -126, -485-3p cluster miR-1, -133a, -126 cluster	Up SA versus controls Up in UA versus controls	Human/53 CAD: 34 SA, 19 UA, 20 controls	Plasma	qRT-PCR	[75]
miR-337-5p miR-145	Up SA versus controls Up in UA versus controls				
					
miR-106b/25 cluster, -17/92a cluster,	Up CAD versus controls	Human/134 CAD: 31 SA, 45 UA, 37 controls	Plasma	qRT-PCR	[156]
-21/590-5p family, -126*, -451					
miR-106b, -25, -92a, -21, -590-5p, -126*	Up UA versus controls	Human/ 5 UA, 5 controls	Plasma MPs	qRT-PCR	
					
miR-340*, -624*	Up	Human/12 premature CAD, 12 controls	Platelets	Array	[77]
		Human/40 CAD, 40 controls	Platelets	qRT-PCR	

ACS: acute coronary syndrome; AP: angina pectoris; CAD: coronary artery disease; CHF: congestive heart failure; CVD: cardiovascular disease; Down: downregulated; miR: microRNA; MPs: microparticles; PBMCs: peripheral blood mononuclear cells; qRT-PCR: quantitative real-time polymerase chain reaction; SA: stable angina; SAP: stable angina pectoris; UA: unstable angina; UAP: unstable angina pectoris; Up: upregulated.

**Table 2:** Circulating miRNAs as diagnostic markers for MI.

miRNAs	Regulation	Groups	Source	Methods	Reference
miR-1	Up	Human/31 STEM, 20 controls	Serum	qRT-PCR	[91]
		Rat/12		Array	
					
miR-1	Up	Human/93 MI, 66 controls	Plasma	qRT-PCR	[92]
					
miR-1, -133, -208b, -499	Up	Human/32 AMI, 36 atypical chest pain,	Plasma	qRT-PCR	[98]
					
miR-1, -133a	Up	Human/29 AMI, 49 non-acute subjects	Serum	qRT-PCR	[112]
					
miR-1, -133a/b, -499-5p	Up	Human/33 MI, 17 controls	Plasma	qRT-PCR	[76]
miR-122, -375	Down	Mouse/5			
					
miR-1, -133a, -208a, -499-5p	Up	Human/25 STEMI, 11 controls Pig/6	Plasma	qRT-PCR	[104]
					
miR-1, -133a, -423-5p, -499-5p, -21	Up	Human/92 NSTEMI, 81 CHF, 99 controls	Plasma	qRT-PCR	[117]
					
miR-1	Down 2-d post-MI, up 5-d post-MI, elevated up to 90-d post-MI	Human/12 post-MI, 12 controls	Plasma	qRT-PCR	[111]
miR-21	Down 2-d post-MI, up 5-d post-MI				
miR-208a, -133a	Up 5-d post-MI, elevated up to 90-d post-MI				
					
miR-1, -133a/b, -208b	Up in STEMI-NSTEMI versus UA	Human/444 ACS (196 STEMI, 131 NSTEMI, 117 UA)	Plasma	qRT-PCR	[105]
					
miR-1, -133a/b	Down	Human/50 MI, 8 controls, 9 fetuses	Heart tissue	qRT-PCR	[100]
miR-208	Up				
					
miR-1, -133, -208b, -499	Up	Human/33 MI, 16 CAD non-MI, 17 others CVD, 30 controls	Plasma	qRT-PCR	[103]
		Rat/18			
					
miR-208a	Overexpression	Mouse/16 Tg, 8 miR-208a^+/+^,	Heart	qRT-PCR	[102]
		10 miR-208a^−/−^, 11 controls			
					
miR-21	Down in infarcted areas; up in border areas	Rat/10	Myocytes	qRT-PCR	[109]
					
miR-21	Up	Rat/12	Heart tissue, myocytes	qRT-PCR	[110]
					
miR-208b, -499	Up	Human/510 MI: 397 NSTEMI, 113 STEMI, 87 controls	Plasma	qRT-PCR	[99]
					
miR-499	Up	Human/14 ACS: 9 IM, 5 AP, 15 CHF; 10 controls	Plasma	qRT-PCR	[97]
					
miR-208	Up (after isoproterenol SC)	Rat/8	Plasma	qRT-PCR	[101]
miR-133, -328	Up	Human/51 AMI, 28 controls	Plasma	qRT-PCR	[120]
					
miR-663b, -1291	Down	Human/20 STEMI, 20 controls	PBMCs	Array	[123]
mirR-30c, -145	Up				
					
miR-423-5p	Up	Human/17 MI, 4 CAD, 5 controls	Plasma	qRT-PCR	[116]
					
miR-133a, -423-5p	Up	Human/246 MI	Plasma	qRT-PCR	[113]
miR-133a	UP AMI versus controls UP CAD versus non-CAD	Human/ 1st cohort:13 AMI, 27 controls; 2nd cohort: 22 CHD, 8 non-CHD; 3rd cohort: 154 CHD, 92 non-CHD	Plasma	qRT-PCR	[115]
					
miR-208b, -320a, -499	Up	Human/224 AMI, 87 controls	Plasma	qRT-PCR	[121]
					
miR-155, -380*	Up	Human/19 dead after 1 y AMI; 21 survival	Serum	qRT-PCR	[122]
					
miR-150	Down	Human/60 AMI	Plasma	Array	[114]
					
miR-1, -133a, -208b, -499	Up	Human/67 AMI, 32 controls	Plasma	qRT-PCR	[96]
					
miR-92a-3p, -30d-5p	Down	Human/13 AMI	Plasma	qRT-PCR	[119]
miR-25-3p, -374b-5p,	Up		Plasma		
miR-186-5p, -342-3p	Down		Platelets		
miR-25-3p, -221-3p	Up		Platelets		
miR-374b-5p	Down		PBMCs		
miR-25-3p, -221-3p	Up		PBMCs		
					
miR-133a	Up	Human/216 AMI with STEMI	Serum	qRT-PCR	[107]
					
miR-1	Up	Human/17 AMI, 25 controls	Plasma	qRT-PCR	[93]
miR-126	Down				
					
miR-30a, 195, -let-7b	Up	Human/18 AMI, 30 controls	Plasma	qRT-PCR	[124]
					
miR-1, -208b, -499-5p	Up	Human/407 MI: 173 STEMI, 146 NSTEMI, 88 non-MI	Plasma	qRT-PCR	[106]

ACS: acute coronary syndrome; AMI: acute myocardial infarction; AP: angina pectoris; CAD: coronary artery disease; CHD: coronary heart disease; CVD: cardiovascular disease; miR: microRNA; d: days; Down: downregulated; HID: heart ischemic disease; MI: myocardial infarction; HF: heart failure; CHF: congestive heart failure; NSTEMI: non-ST elevation myocardial infarction; PBMCs: peripheral blood mononuclear cells; qRT-PCR: quantitative real-time polymerase chain reaction; SA: stable angina; SC: subcutaneously; STEMI: ST elevation myocardial infarction; Tg: transgenic; UA: unstable angina; Up: upregulated; y: year.

**Table 3:** Circulating miRNAs as diagnostic markers for HF.

miRNAs	Regulation	Groups	Source	Methods	Reference
miR-24, -125b, -195, 199a, -214	Up	Human/6 HF, 4 controls	Left ventricle	Northern blot	[131]
miR-24, -125b, -195, 199a, -214	Up	Mice/3 Tg, 3 controls	Heart	Array, qRT-PCR	
					
miR-214	Up	Human/19 ICM, 25 DCM, 13	Left ventricle	qTR-PCR	[11]
miR-19a/b	Downin DCM-AS	AS, 10 controls		Bead-based hybridization, qTR-PCR	
					
miR-21, -129, -212	Up in fetal heart	Human/6 HF, 6 fetal hearts, 4 controls	Heart	Array, qRT-PCR	[130]
					
miR-100, -195	Up	Human/6 IDC, 5 ICM,	Heart	Array, qRT-PCR	[132]
miR-92, -133b	Down	6 controls			
					
miR-125, -181b, -214, -342	Up	Human/50 end-stage HF	Left ventricle	Array	[126]
miR-1, -7, -29b, -378	Down	(DCM), 20 controls			
					
32 miRs	Up	Human/17 HF, 10 post-LVAD, 10 controls	Heart	Array	[135]
					
miR-126	Up	Human/33 IHD (MI, UA, SA),	Plasma	qRT-PCR	[141]
miR-122, -499	No variation	10 HF, 17 controls			
miR-126, -130a	Down	Human/45 CHF (ICM), 15 CHF (DCM), 35 controls	Angiogenic EOCs, CD34^+^	Array, qRT-PCR	[142]
		Mice/8-16			
					
miR-126	Down	Human/106 CHF: 55 ICM, 51 NICM; 30 controls	Mononuclear cells	Array	[145]
miR-508-5p	Up				
					
miR-423-5p, -18*, -129-5p, miR-622, HS_202.1, -1254	Up	Human/30 HF, 20 HF with dyspnea, non-HF with dyspnea, 39 controls	Plasma	qRT-PCR	[118]
					
miR-423-5p, -320a, -22, -92b	Up	Human/30 chronic HF, 30 controls	Serum	qRT-PCR	[138]
					
miR-423-5p	No variation	Human/41 right ventricular HF, 10 controls	Plasma	qRT-PCR	[140]
					
miR-423-5p	Up	Human/45 DCM, 39 controls	Plasma	qRT-PCR	[139]
					
miR-103, -142-3p, -30b, -342-3p	Down	Human/32 HF, 15 COPD, 14 controls	Plasma	qRT-PCR	[137]
					
miR-499	Up	Human/33 HF, 34 controls	Plasma	qRT-PCR	[98]
					
miR-499	No variation	Human/15 CHF, 10 controls	Plasma	qRT-PCR	[97]
					
miR-107, -139, and -142-5p	Down in DCM and NIDCM	Human/15 ICM, 19 NCDCM, 19 controls	PBMCs	qRT-PCR	[144]
miR-142-3p and -29b	Up in NIDCM				
miR-125b and -497	Down in DCM				
					
miR-210	Up	Human/39 HF	Plasma	qRT-PCR	[146]
miR-210	Up	Human/13 CHF: 8 NYHA II, 5 NYHA III and IV; 6 controls	Mononuclear cells	qRT-PCR	
miR-15a, -15b, -20a, -103, -130a, -130b, -195, -210, -301b, -451, -494	Up	Rat/13HF, 9 controls	Plasma	Array	
					
miR-210, -30a	Up	Human/22 HF, 18 controls, 9 fetuses	Serum	qRT-PCR	[147]
					
miR-133a	Up	Human/74 AS	Plasma, LV biopsies	qRT-PCR	[153]
					
miR-21	Up	Human/75 AS, 32 surgical patients, 25 controls	Plasma, LV biopsies	qRT-PCR, in situ	[154]
				hybridization	
					
miR-122	Down	Human/28 AS: 15 SF, 13 non-SF; 10 controls	Endomyocardial biopsies	qRT-PCR	[155]

AP: angina pectoris; AS: aortic stenosis; CHF: chronic heart failure; COPD: chronic obstructive pulmonary disease; DCM: dilated cardiomyopathy; Down: downregulated; EOCs: early outgrowth cells; HF: heart failure; ICM: ischemic cardiomyopathy; IDC: idiopathic cardiomyopathy; IHD: ischemic heart disease; ISC: ischemic cardiomyopathy; LVAD: left ventricular assist device; MI: myocardial infarction; miR: microRNA; NICM: nonischemic cardiomyopathy; NIDCM: nonischemic dilated cardiomyopathy; non-SF: nonsevere fibrosis; NYHA: New York Heart Association; qRT-PCR: quantitative real-time polymerase chain reaction; SA: stable angina; SF: severe fibrosis; UA: unstable angina; Up: upregulated.

## List of Acronyms

ACE: Angiotensin-converting enzyme
ACS: Acute coronary syndrome
ADAR: Adenosine deaminases acting on RNA
Ago: Argonaute
AMI: Acute myocardial infarction
AP: Angina pectoris
AS: Aortic stenosis
ASO: Antisense-based oligonucleotide
AVR: Aortic valve replacement
BCL2L11: B-cell leukemia/lymphoma-2 like protein 11
BIM: BCL2-like 11
CAD: Coronary artery disease
CaM: Calmodulin
CaMKII*γ*: Ca^2+^/CalModulin-dependent protein kinase II *γ*
CHD: Coronary heart disease
CHF: Congestive heart failure
CK-MB: Creatine kinase-MB
CLCF1: Cardiotrophin-like cytokine factor 1
CnA: Calcineurin A
COPD: Chronic obstructive pulmonary disease
cTnI: Cardiac troponin-I
CVD: Cardiovascular disease
CYPD: Cyclophilin D
DCM: Dilated cardiomyopathy
DGCR8: DiGeorge syndrome critical gene 8
ECs: Endothelial cells
EMB: Endomyocardial biopsy
EOCs: Early outgrowth cells
EPCs: Endothelial progenitor cells
ERBB2: Receptor tyrosine-protein kinase erbB-2
Exp 5: Exportin-5
FGF: Fibroblast growth factor
HDAC4: Histone deacetylase 4
HDGF: Hepatoma-derived growth factor
HIF-1*α*: Hypoxia-induced factor-1*α*
HF: Heart failure
HOP: Homeodomain-only protein
hs-cTnT: High-sensitivity cardiac troponin T
HOXA5: Homeobox A5
ICM: Ischemic cardiomyopathy
IDC: Idiopathic cardiomyopathy
IHD: Ischemic heart disease
ISC: ISchemic cardiomyopathy
ISCU: Iron-sulfur clusters assembly protein
LAD: Left anterior descending
LV: Left ventricle
LVB: Left ventricle biopsy
MEF2A: Myocyte-specific enhancer factor 2A MEF2A
miRISC: miRNA-induced silencing complex
miRNAs/mIRs: microRNAs
LLT: Lipid lowering therapy
LNA: Locked nucleic acid
LVM: Left ventricular mass
MAP: Mitogen-activated protein
MI: Myocardial infarction
MPs: Microparticles
MOE: 2′-*O*-Methoxyethyl
MREs: miRNA recognition elements
NCX1: Sodium-calcium exchanger 1
NELF-A/WHSC2: Negative elongation factor A/Wolf-Hirschhorn syndrome
NGS: Next generation sequencing
non-SF: Nonsevere fibrosis
NPM1: Nuclephosmin 1
NSTEMI: Non-ST elevation myocardial infarction
NT-proBNP: N-terminal probrain natriuretic peptide
NYHA: New York Heart Association
Omes: 2′-*O*-Methyl modified oligonucleotides
ORF: Open reading frame
PCR: Polymerase chain reaction
PCR-RLFP: PCR-based restriction fragment length polymorphisms
PE: Phenylephedrine
PIK3: Phosphatidylinositol 3-kinase
PBMCs: Peripheral blood mononuclear cells
PHLPP1: PH domain leucine-rich repeat protein phosphatase
PTEN: Phosphatases TENsin homolog
qRT-PCR: Quantitative real-time polymerase chain reaction
RISC: RNA-induced silencing complex
SA: Stable angina
SAP: Stable angina pectoris
SF: Severe fibrosis
SIRT1: SIRTuin 1
SLC2A: Solute carrier family 2A
SPRED1: Sprouty-related EVH1 domain-containing protein 1
STEMI: ST elevation myocardial infarction
TAC: Transverse aortic constriction
Tg: Transgenic
TGF-b1: Transforming growth factor beta 1 (TGF-b1)
THRAP1: Thyroid hormone receptor associated protein 1
TRBP: TAR RNA binding protein
XRN-2: 5′-to-3′ exoribonuclease 2
UA: Unstable angina
UAP: Unstable angina pectoris
3′-UTR: 3′-Untranslated region
5′-UTR: 5′-Untranslated region
VCAM1: Vascular cell adhesion molecule 1
VEGF: Vascular endothelial growth factor.
